# Morphology and Comparative Transcriptome Analysis of Resistant and Susceptible Bitter Gourd (*Momordica charantia* L.) Reveals the Molecular Response Related to Powdery Mildew Resistance

**DOI:** 10.3390/jof12010080

**Published:** 2026-01-22

**Authors:** Lei Xia, Kai Wang, Feng Guan, Bo Shi, Xuetong Yang, Yuanyuan Xie, Xinjian Wan, Jingyun Zhang

**Affiliations:** 1 Institute of Vegetables and Flowers, Jiangxi Academy of Agricultural Sciences, Nanchang 330200, China; 2 Jiangxi Key Laboratory of Horticultural Crops (Fruit, Vegetable & Tea) Breeding, Jiangxi Academy of Agricultural Sciences, Nanchang 330200, China; 3 Jiangxi Engineering Research Center of Vegetable Molecular Breeding, Jiangxi Academy of Agricultural Sciences, Nanchang 330200, China

**Keywords:** powdery mildew, bitter gourd, transcriptome, differentially expressed genes, transcription factor, *MLO*

## Abstract

Powdery mildew (PM) is a major disease affecting bitter gourd cultivation, and resolving the molecular regulatory mechanisms underlying PM resistance is important for bitter gourd molecular breeding for resistance. In this study, morphological and molecular methods were used to identify the PM pathogen in bitter gourd, and comparative transcriptome analysis was performed on leaves of the resistant cultivar R and the susceptible cultivar S after PM infection. The morphological and molecular identification results showed that the PM pathogen in bitter gourd was *Podosphaera xanthii*. Scanning electron microscopy results revealed that the *P. xanthii* exhibited distinct growth patterns in the R and S after *P. xanthii* infection. Compared to the S, the R exhibited 3966, 2729, 5891, and 3878 differentially expressed genes (DEGs) at 0, 2, 3, and 4 days after *P. xanthii* infection, respectively. KEGG enrichment analysis showed that DEGs were primarily enriched in plant–pathogen interactions, MAPK signaling pathway plants, and plant hormone signal transduction pathways. Transcription factor (TF) analysis of differentially expressed genes revealed that *MYB*, *bHLH*, and *ERF* family members could be involved in the defense process against the *P. xanthii* infection. Moreover, the analysis of the *MLO* genes revealed that *Moc10g30350.1* could be involved in regulating PM resistance. These findings could enrich the molecular theoretical basis for resistance to PM, and provide new insights for the molecular breeding process of bitter gourd resistance to PM.

## 1. Introduction

Powdery mildew (PM) is a common fungal disease, frequently occurring in crops such as rice [1], wheat [2], peppers [3], grapes [4], and melons [5], which is important for crop yield and quality [6]. PM typically begins on lower, older leaves, which causes the appearance of small, circular, white, powdery fungal spots on the leaf surface [7]. These spots could affect photosynthesis in plant leaves, reducing the crop yield and quality. Early microscopic studies revealed that the leaf spots caused by PM were fungal colonies, which were composed of fungal mycelium and conidiophores [8]. PM is one of the common diseases in cucurbit plants, which limits the high-quality production of cucurbit crops [9]. It is shown that PM in cucurbit crops is primarily caused by the biotrophic fungi *Podosphaera xanthii* and *Golovinomyces cichoraceum* [10]. Among these, *Pdosphaera xanthii* was found to be predominant in tropical and subtropical regions, while *G. cichoracearum* dominated in temperate zones [11]. Due to climatic and geographical variations, *P. xanthii* has evolved multiple races, such as races 1–3 and S in the USA [12]. Owing to the pathogen’s high genetic variability and adaptability, variety resistance could lose. Therefore, identifying PM physiological races and understanding the molecular mechanisms of host resistance could be important for PM resistance breeding.

There are two immune systems in plants against PM infection: pathogen-associated molecular pattern-induced immunity (PTI) and effector-induced immunity (ETI) [13]. As the first line of defense, PTI could be initiated by pattern recognition receptors (PRRs) on the plant surface and within cells, including *FLS2* [14] and *EFR* [15]. It has been revealed that PTI could regulate PM resistance by activating the MAPK cascade, inducing Ca^2+^ influx, and triggering RBOHD-mediated ROS bursts [16,17]. Among these, transcription factors such as *bHLH* [18], *MYB* [19], and *WRKY* [20] could participate in the PTI process, and consequently regulate the expression of PM resistance genes in plants. For example, in grapes, *VqWRKY56* has been shown to regulate resistance to PM, and overexpression of *VqWRKY56* could enhance PM resistance through ROS accumulation [21]. Furthermore, it has been found in multiple species that *MLO* genes are widely involved in plant growth, development, and stress responses: particularly PM resistance [22]. Silencing or knocking out specific *MLO* genes could confer PM resistance in plants. For example, knocking out four genes (*Gmmlo02*, *Gmmlo19*, *Gmmlo20*, and *Gmmlo23*) could significantly enhance soybean resistance to PM [23].

Bitter gourd (*Momordica charantia* L.) belongs to the *Cucurbitaceae*, which is a vegetable crop with significant medicinal value, such as anti-diabetic, anti-obesity, antioxidant, and blood lipid-lowering properties [24,25,26]. Bitter gourd is mainly cultivated in tropical and subtropical regions, where high humidity and temperature often cause PM [27]. The occurrence of PM resulted in leaf disease, affecting photosynthesis and reducing the yield of bitter gourd. In bitter gourd cultivation, chemical control was the main approach to managing PM, but it led to increased production costs and environmental pollution. Developing resistant bitter gourd varieties through molecular selective breeding is one important strategy for controlling PM occurrence. Uncovering the molecular mechanisms’ underlying resistance to PM and identifying key genes involved in the response to PM could be important for molecular breeding of PM resistance in bitter gourd.

With the rapid advancement of next-generation sequencing (NGS) technology, transcriptomics has been widely applied in studying the defense genes and molecular regulatory mechanisms involved in the response to plant pathogens [28]. For example, through the transcriptome analysis, Zhang et al. found pathways including starch and sucrose metabolism, photosynthesis, and fatty acid metabolism, which could be involved in the PM resistance process of ‘BlackJack’ [29]. Liu et al. conducted transcriptome analysis, and found that the phenylpropanoid biosynthesis and flavonoid biosynthesis pathways were involved in the PM resistance from inbred line ‘63187’ [30]. Ba et al. identified that allantoin and jasmonic acid could mediate the resistance response to PM in melon [31].

In this study, morphological and molecular biological identification of PM pathogens in bitter gourd leaves were conducted. The differences in leaf morphology between resistant materials, R, and susceptible materials, S, at different periods after PM infection were investigated by using scanning electron microscopy. Moreover, transcriptome analysis was conducted to identify differentially expressed genes (DEGs) and key pathways involved in the PM response. These findings could contribute to understanding the molecular mechanisms underlying bitter gourd resistance to PM, which would provide a theoretical basis for developing PM-resistant cultivars in bitter gourd.

## 2. Materials and Methods

### 2.1. Identification and Phylogenetic Analysis of PM Pathogen

The leaves of bitter gourds that were naturally infected with PM were collected. Following the method of Duan et al. [32], the PM pathogen was purified. A single white spot was selected and placed in 0.5 mL of 0.3% KOH solution. The mixture was spotted onto a microscope slide and observed under an optical microscope (Olympus, Tokyo, Japan) to investigate the morphology of the PM pathogen.

For molecular identification of the PM pathogen, ITS primers (Table S1) were used to PCR amplify the ITS sequence from the bitter gourd PM pathogen. The PCR program was 94 °C for 5 min, followed by 35 cycles of 94 °C for 30 s, 55 °C for 30 s, and 72 °C for 45 s, an amplification of 72 °C for 10 min, and final storage at 4 °C. The PCR reaction volume was 20 μL, comprising 2 μL DNA template, 1 μL F primer, 1 μL R primer, 10 μL PrimeSTAR^®^ Max DNA Polymerase high-fidelity DNA polymerase, and 6 μL ddH_2_O. Moreover, sequences were downloaded from the NCBI database for *P. xanthii*, *G. cichoracearum*, *Erysiphe polygoni*, and *Leveillula taurica* to perform phylogenetic analysis. Multiple sequence alignment was performed by DNAMAN (Lynnon Corporation, Vaudreuil-Dorion, QC, Canada), and a phylogenetic tree was constructed using MAGE v11 software [33], based on the neighbor–neighbor method.

### 2.2. Plant Materials and Treatment

The PM-resistant variety, R, and susceptible variety, S, of bitter gourd were preserved in the Vegetables and Flowers Research Institute of the Jiangxi Academy of Agricultural Sciences. After germination, seeds were sown in 32-cell trays, and soil was prepared with a mixture of peat, perlite, and fertilizer at a ratio of 6:3:1. Subsequently, they were placed in a plant-growth room, where the environment was set to 26 °C for 14 h light and 20 °C for 10 h dark. When seedlings reached the two-leaf stage, 1 × 10^6^/mL spore suspension was prepared and sprayed onto bitter gourd leaves using a spray bottle. Leaf samples were collected at 0 d, 2 d, 3 d, and 4 d after PM inoculation, rapidly frozen in liquid nitrogen, and stored at −80 °C. Three biological replicates were taken at each period.

### 2.3. Scanning Electron Microscope

Leaf samples were collected at 0 d, 2 d, 3 d, and 4 d after PM inoculation, cut into 5 mm pieces, and placed in the electron microscope fixative (Glutaraldehyde, 2.5% (EM Grade)). Samples were then fixed at 4 °C for at least 24 h. The samples were then washed three times with 0.1 M phosphate buffer (pH = 7.0), followed by immersion in tert-butanol mixtures containing 50%, 70%, 80%, and 90% ethanol for 1 h each. Finally, samples underwent freeze-drying and gold sputtering. Finally, PM fungi on the leaves of each material were microscopically observed using a scanning electron microscope SU8100 (HITACHI, Tokyo, Japan).

### 2.4. RNA-Seq Analysis

Total RNA was extracted using the Trizol from the leaves of PM-resistant variety R and susceptible variety S after PM inoculation with 3 replications. The concentration and integrity of RNA was accurately detected by NanoDrop 2000 spectrophotometer (Thermo Fisher Scientific, Waltham, MA, USA) and Agilent 2100 Bioanalyzer (Agilent Technologies, Santa Clara, CA, USA). All RNA samples exhibited RIN values ≥ 6, which could meet sequencing requirements. The mRNA of each library was sequenced on the MGI sequencing platform, located at Wuhan Benagen Tech Solution Co., Ltd. (Wuhan, China; http://www.benagen.com). The raw data were quality-controlled by Fastqc [34]. The reference genome was downloaded from bitter gourd (OHB3-1 v2) from the Cucurbit Genome Database version 2 (http://cucurbitgenomics.org/v2/, accessed on 7 January 2026). The clean data were aligned to the reference genome by Hisat2 v2.2.1 [35]. The expressions of individual genes were counted by the featureCounts [36]. Expression values were normalized by converting them to fragments per million (FPKM). Leaf samples from susceptible bitter gourd S plants at 0 days, 2 days, 3 days, and 4 days after *P. xanthii* infection were used as controls. DESeq2 v1.34.0 [37] was used to find the differentially expressed genes (DEGs). DEGs identification employs FDR correction via the Benjamini–Hochberg method, filtering results based on FDR < 0.05 and |log_2_FC| ≥ 1 to strictly control false positives.

### 2.5. Analysis of the MLO Gene

To obtain the *MLO* gene from bitter gourd, the hidden Markov model (HMM) for the MLO protein domain (PF03094) was downloaded from the Pfam database. The bitter gourd (OHB3-1 v2) protein sequences were downloaded from the Cucurbit Genome Database version 2 (http://cucurbitgenomics.org/v2/, accessed on 7 January 2026). The HMMER3.0 search tool from Tbtools [38] was used to identify *MLO* genes in bitter gourd. The candidate Mlo protein domains were further checked by the NCBI conserved structural domain database (NCBI-CDD, https://www.ncbi.nlm.nih.gov/cdd/). Based on the above transcriptome data analysis results, the expression levels of *MLO* genes at different periods in time were plotted by TBtools software [38].

### 2.6. qRT-PCR Analysis

The primers for qRT-PCR (Table S1) were used by the Primer-BLAST program in NCBI. *Moc02g21040.1* (Actin) was selected as the reference gene. qRT-PCR was performed in a 96-well plate, using a BIO-RAD CFX96 Touch Real-Time PCR Detection System (Applied Biosystems, San Francisco, CA, USA) with SYBR Green PCR Master Mix. The qRT-PCR amplification was 94 °C for 10 min, followed by 40 cycles of 94 °C for 5 s and 65 °C for 30 s. The gene expression level was calculated by 2^−ΔΔCt^ [39] with three biological and three technical replicates.

### 2.7. Statistical Analysis

The result for each sample is shown as mean ± standard deviation (SD) from three replicates, which was analyzed by Excel. A two-tailed Student’s *t*-test was used to analyze the significance of differences. *p* value < 0.05 (*) and *p* value < 0.01 (**) were regarded as significant.

## 3. Results

### 3.1. Identification of PM Pathogen in Bitter Gourd Leaves

**Figure 1:**
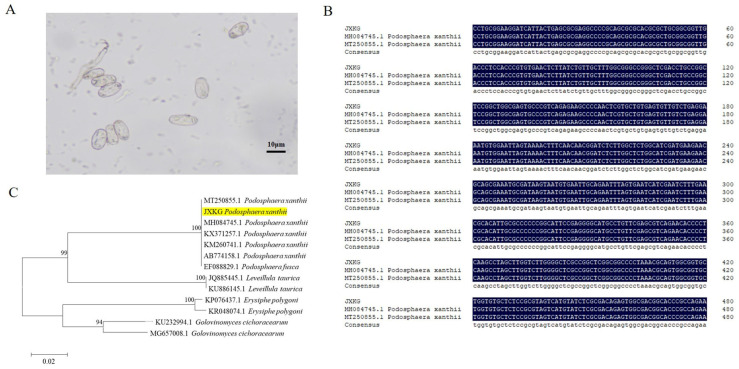
Morphological and molecular identification of PM pathogen in bitter gourd leaves. (**A**) Morphological observations of PM pathogen in bitter gourd leaves, scale bar = 10 μm; (**B**) sequence alignment of ITS sequence in bitter gourd PM, pumpkin (MT250855.1), and squash (MH084745.1); and (**C**) phylogenetic tree analysis of ITS sequence of *P. xanthii*, *G. cichoracearum*, and *E. polygoni*.

To investigate the type of PM pathogen in bitter gourd, morphological observations and molecular identification of the PM pathogen were conducted. The microscopic observation results revealed that the conidia of the bitter gourd PM pathogen were all ellipsoidal, colorless, unicellular, and well-developed hyphae (Figure 1A), which could be identified as PM fungus of cucurbits. ITS sequence analysis revealed that the PM pathogen sequence from bitter gourd was identical to *P. xanthii* sequences from pumpkin (MT250855.1) and squash (MH084745.1) (Figure 1B), which were *P. xanthii*. Phylogenetic analysis results showed that the PM pathogen sequence from bitter gourd clustered with *P. xanthii* sequences from pumpkin (Figure 1C), which proved that the PM pathogen in bitter gourd was *P. xanthii*.

### 3.2. Phenotypic Changes and Microscopic Observation in Bitter Gourd Leaves After P. xanthii Infection

**Figure 2:**
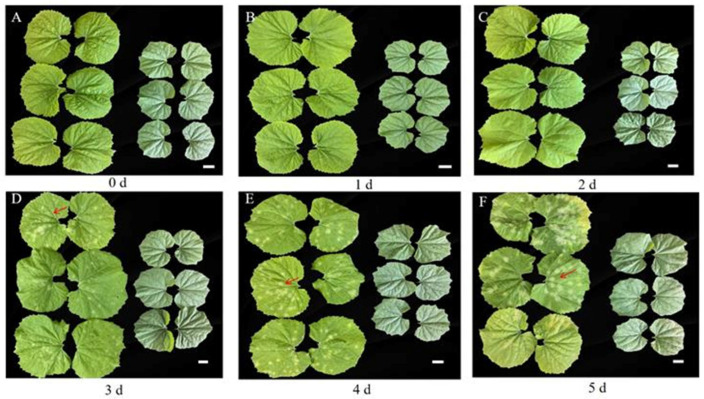
Phenotypic changes in resistant cultivar R and the susceptible cultivar S leaves after *P. xanthii* infection. (**A**–**F**) Leaf characteristics of R and S materials at 0 d (**A**), 1 d (**B**), 2 d (**C**), 3 d (**D**), 4 d (**E**), and 5 d (**F**) after *P. xanthii* infection; scale bar = 2 cm. In each image, the left side was for the S material, and the right side was for the R material. Arrows showed PM on leaves.

To understand the dynamic changes in *P. xanthii* infection of bitter gourd leaves, the phenotypes of bitter gourd R and S materials were observed and compared at different periods after *P. xanthii* infection. The results showed that there was no significant phenotypic difference between R and S material plants at 0–2 days after infection (Figure 2A–C). On the third day, S material leaves exhibited small white spot sand and PM infection symptoms, while R material leaves remained without white spots (Figure 2D). At 4–5 days, PM symptoms progressively worsened on S material leaves, and the white spots on leaves increased (Figure 2E,F). However, the R material still showed no obvious disease symptoms at 4–5 days. Therefore, it is concluded that the R material exhibits obvious resistance to PM.

**Figure 3:**
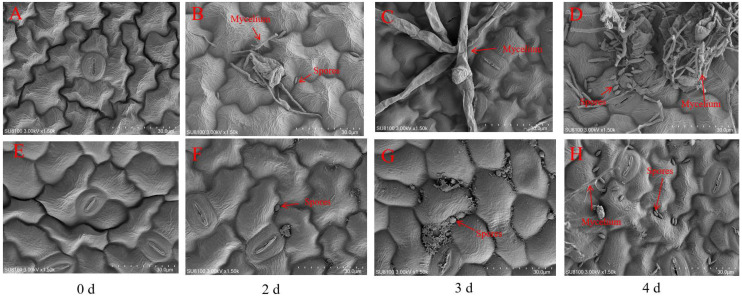
Scanning electron microscopy (SEM) observations in resistant cultivar R and the susceptible cultivar S leaves after *P. xanthii* infection. (**A**–**D**) SEM observations in leaf characteristics of S materials at 0 d (**A**), 2 d (**B**), 3 d (**C**), and 4 d (**D**) after *P. xanthii* infection and (**E**–**H**) SEM observations in leaf characteristics of R materials at 0 d (**E**), 2 d (**F**), 3 d (**G**), and 4 d (**H**) after *P. xanthii* infection.

Additionally, scanning electron microscopy (SEM) observations were conducted on R and S leaves at 0 d, 2 d, 3 d, and 4 d after *P. xanthii* infection. The results showed that at 0 d, R and S leaf cells appeared smooth with clearly visible stomata and no hyphal growth (Figure 3A,E). As the infection time progressed, S leaves exhibited minor hyphal growth on the second day (Figure 3B), while only 1–2 spores were observed in R leaves (Figure 3F). On the third day, abundant hyphae were observed in the S material (Figure 3C), while only a few spores were noted in the R material (Figure 3G). On the fourth day, when PM erupted in the S material, a large number of spores and mycelium were clearly visible (Figure 3D). However, only a few spores and minimal mycelium were observed in the R material (Figure 3H). Therefore, it is considered that R should exhibit marked resistance to PM.

### 3.3. Transcriptomic Data Analysis of Bitter Gourd Leaves After P. xanthii Infection

**Figure 4:**
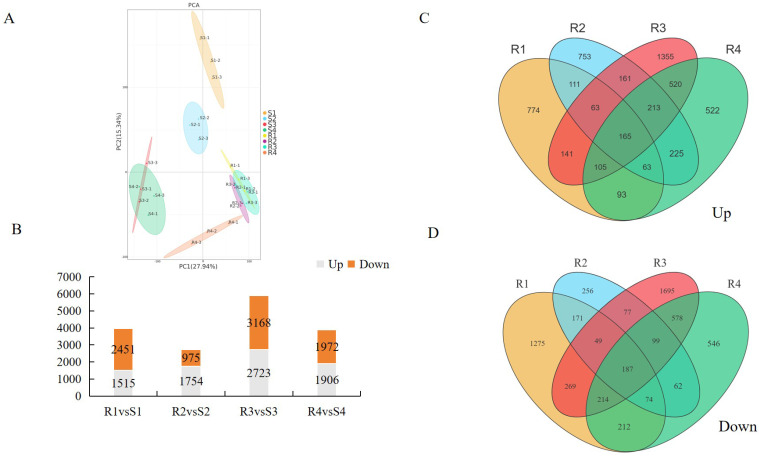
Analysis of transcriptomic data in R and S leaves after *P. xanthii* infection. Among these, S for S-material, while R for R-material. (**A**) PCA of transcriptomic data; (**B**) DEGs analysis of transcriptomic data; (**C**) Venn diagrams of the upregulated DEGs; and (**D**) Venn diagrams of the downregulated DEGs.

To reveal the molecular mechanisms underlying the differing resistance to PM between R and S, RNA-seq technology was employed to investigate the gene expression profiles at 0 d, 2 d, 3 d, and 4 d after *P. xanthii* infection. Each sample was subjected to three biological replicates, and a total of 24 cDNA libraries were generated (Table 1). Each library obtained an average of 6.28 Gb of clean reads, and the Q30% exceeded 97.80% (Table 1). Approximately 89.43% to 97.43% of clean reads mapped to the bitter gourd reference genome, where 80.98% to 93.32% mapped uniquely and 1.97% to 10.58% mapped to multiple locations (Table 1). The principal component analysis (PCA) results revealed high replication among samples within R and S (Figure 4A).

To analyze the molecular and biological functions of DEGs, GO and KEGG enrichment analyses, the analysis of differential expression genes (DEGs) was performed using log_2_|FoldChange| ≥ 1 and *p* value < 0.05 as parameters between different samples (Tables S2–S5). The results for DEGs at different periods showed that compared to the S material, R contained 3966 DEGs (1515 upregulated genes and 2451 downregulated genes) (Table S2), 2729 DEGs (1754 upregulated and 975 downregulated) (Table S3), 5891 DEGs (2723 upregulated and 3168 downregulated) (Table S4), and 3878 DEGs (1906 upregulated and 1972 downregulated) (Table S5), respectively (Figure 4B). Among these, the highest number of DEGs occurred on the third day, which was the powdery mildew attack stage in the S material. Cluster analysis of DEGs revealed that in the four stages, 165 genes were significantly upregulated in the R material (Figure 4C), while 187 genes were significantly downregulated in the R material (Figure 4D).

### 3.4. GO and KEGG Enrichment Analysis of DEGs

**Figure 5:**
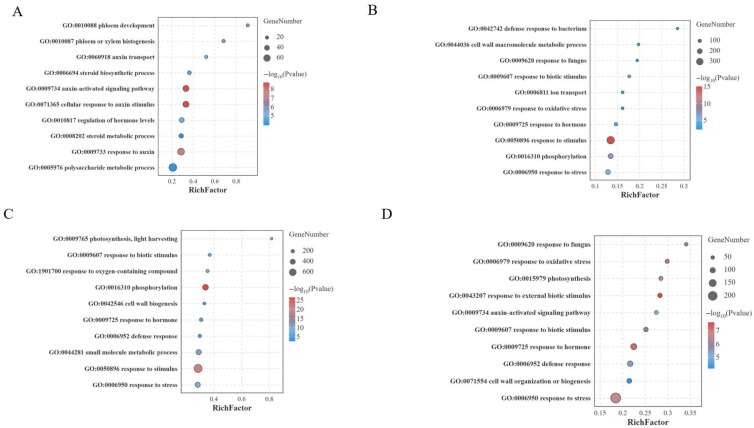
GO enrichment analysis of DEGs between R and S leaves after *P. xanthii* infection. (**A**–**D**) GO enrichment for biological process of DEGs at 0 d (**A**), 2 d (**B**), 3 d (**C**), and 4 d (**D**) after *P. xanthii* infection.

To analyze the molecular and biological functions of DEGs, GO and KEGG enrichment analyses were performed. GO analysis revealed that at 0 days, DEGs were primarily enriched in auxin-related pathways (auxin-activated signaling pathway (GO:0009734), auxin transport (GO:0060918), and response to auxin (GO:0009733)) and steroid metabolism processes (GO:0008202) (Figure 5A). During the period of *P. xanthii* infection, the DEGs were predominantly enriched in multiple stress-related GO pathways, including response to stimulus (GO:0050896), response to stress (GO:0006950), response to biotic stimulus (GO:0009607), defense response (GO:0006952), and cell wall biosynthesis (GO:0042546) (Figure 5B–D). As PM spread incessantly in susceptible material S, photosynthesis in leaves was also affected. On the third and fourth days, DEGs were enriched in GO pathways related to plant photosynthesis, such as photosynthesis, light harvesting (GO:0009765), and photosynthesis (GO:0015979) (Figure 5C,D).

**Figure 6:**
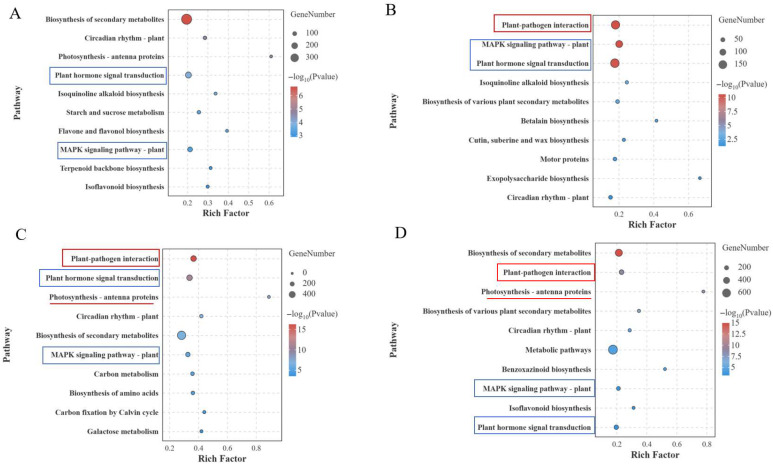
KEGG enrichment analysis of DEGs between R and the S leaves after *P. xanthii* infection. (**A**–**D**) KEGG enrichment of DEGs at 0 d (**A**), 2 d (**B**), 3 d (**C**), and 4 d (**D**) after *P. xanthii* infection.

The KEGG enrichment results showed that DEGs were enriched in two pathways at four periods, including plant hormone signaling transduction pathways and the MAPK signaling pathway plant (Figure 6). In plant hormone signaling transduction pathways, JA-related genes (*Moc01g33300.1* and *Moctig17757g030.1*) were upregulated, and auxin-related genes (*Moc06g25830.1* and *Moc03g03590.1*) were downregulated in R as powdery mildew inoculation period increased (Figure S1), which implied that plant hormones might be involved in regulating plant disease resistance. Furthermore, the results were validated by qRT-PCR (Figure S1). As the inoculation of PM progressed, DEGs were significantly enriched in plant–pathogen interaction pathways on the second, third, and fourth days (Figure 6B–D). As the *P. xanthii* spread on the S material, the DEGs also showed partial enrichment in photosynthesis–antenna proteins–plants at third and fourth days (Figure 6C,D). Collectively, these findings reveal distinct genetic and pathway differences in the resistant and susceptible material after *P. xanthii* infection.

**Figure 7:**
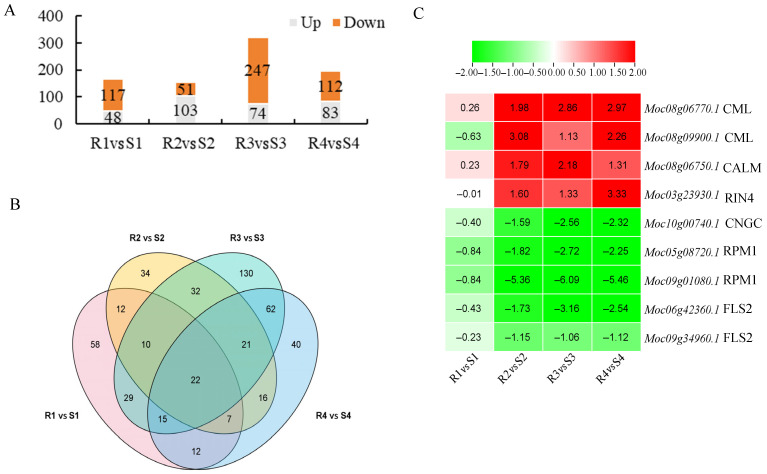
Analysis of plant–pathogen interaction pathway and heat map of DEGs in R and S responding to *P. xanthii* infection. Among these, S for S-material, while R for R-material. (**A**) Statistics of DEGs in plant–pathogen interaction pathway; (**B**) Venn diagrams of DEGs; and (**C**) heat map of DEGs in plant–pathogen interaction pathway responding to *P. xanthii* infection. The values in the heatmap represent the log_2_FoldChange values of genes in R compared to S.

Plant–pathogen interactions are associated with plant defense mechanisms. In this study, significant divergence was observed between R and S materials in the plant–pathogen interaction pathway after *P. xanthii* infection. The 190, 159, 309, and 179 DEGs were identified in the plant–pathogen interaction pathway at 0 d, 2 d, 3 d, and 4 d, respectively (Figure 7). Cluster analysis results showed that 14 DEGs were present on the second, third, and fourth days after *P. xanthii* infection (Figure 7B). Among these, calcium-binding proteins (*CML*, *Moc08g06770.1* and *Moc08g09900.1*) and calmodulin (*CaM*, *Moc08g06750.1*) exhibited significant upregulation in R leaves. Conversely, serine/threonine protein kinase (*FLS2*, *Moc06g42360.1*, and *Moc09g34906.1*), plant immune receptor protein (*RPM1*, *Moc05g08720.1* and *Moc09g01080.1*), and cyclic nucleotide-gated channels (*CNGCs*, *Moc10g00740.1*) exhibited significant downregulation in R leaves (Figure 7C). These findings implied that the defense response of R material against *P. xanthii* could be guided by *CaM*/*CML*-mediated signal transduction. Interestingly, we found that at 0 days, compared to S material, R material exhibited a downregulated expression of genes associated with *FLS2*, while genes related to *CML* were upregulated (Figure S2). These results proved that R and S materials themselves exhibit significant differences in the expression of disease resistance genes in the plant–pathogen interaction pathway, which lead to differences in plant PM resistance.

### 3.5. TF Analysis Involved in Regulation of PM Resistance

**Figure 8:**
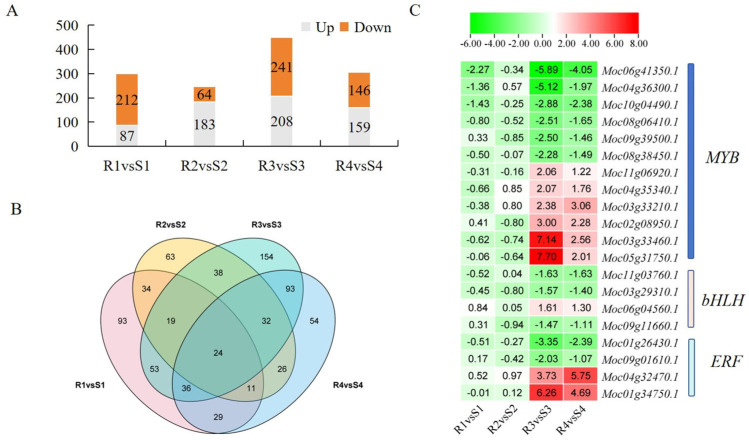
TF analysis and heat map of DEGs in R and S responding to *P. xanthii* infection. (**A**) Statistics of differentially expressed TFs; (**B**) Venn diagrams of differentially expressed TFs; and (**C**) heat map of differentially expressed TFs in R and S responding to *P. xanthii* infection. The values in the heatmap represent the log_2_FoldChange values of genes in R compared to S.

Transcription factors (TFs) have an important role in regulating plant disease resistance. In this study, 299, 247, 449, and 305 differentially expressed TFs were identified at 0 d, 2 d, 3 d, and 4 d, respectively (Figure 8A). Among them, the highest number of differentially expressed TFs occurred on the third day after *P. xanthii* infection, including 208 upregulated TFs and 241 downregulated TFs. Cluster analysis of differentially expressed TFs revealed that 24 TFs were present in all four periods (Figure 8B), including *B3* (1), *bHLH* (2), *bZIP* (2), *C2H2* (1), *C3H* (4), *HD*-ZIP (1), *HSF* (2), *MADS* (2), *MYB* (2), *RAV* (1), *SBP* (2), *SBP* (1), *Trihelix* (2), and *WRKY* (1). As PM disease progressed in the S material, the number of differentially expressed TFs gradually increased. On the third and fourth days, 93 uniquely present differentially expressed TFs were identified. Analysis of these 93 TFs revealed that they primarily belonged to three categories—*MYB*, *bHLH*, and *ERF* TFs (Figure 8C)—which might be involved in the PM resistance process of R material.

### 3.6. MLO Genes Related to PM Resistance

**Figure 9:**
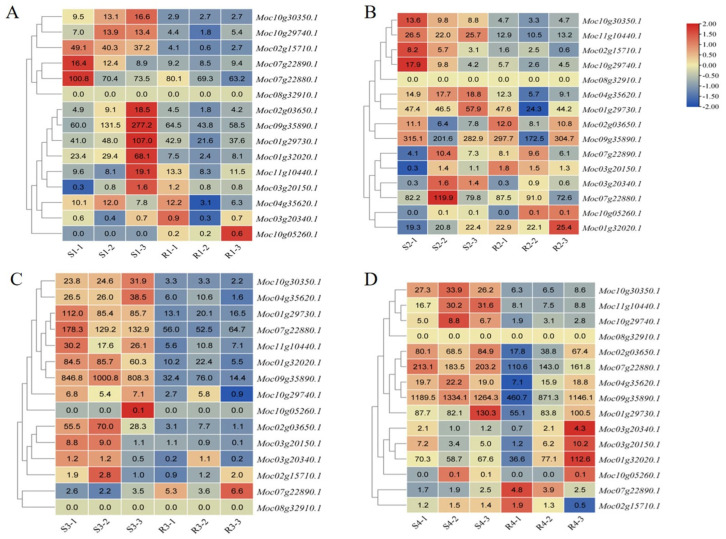
Heat map analysis of *MLO* genes in R and S responding to *P. xanthii* infection. (**A**–**D**) Heat map analysis of *MLO* genes at 0 d (**A**), 2 d (**B**), 3 d (**C**), and 4 d (**D**) after *P. Xanthi* infection. The values in the heatmap represent the FPKM values of the genes.

MLO genes play a crucial role in plant resistance to PM. In this study, a total of 15 *MLO* genes were identified in bitter gourd (Figure 9). At the 0 d stage, compared to the S material, *MLO* genes were generally downregulated in the R material, including significantly down-expressed genes such as *Moc10g30350.1*, *Moc02g15710.1*, and *Moc01g32020.1* (Figure 9A). As *P. xanthii* infection progressed in leaves, transcription levels of *MLO* genes in the R material showed significant reduction. On the third day, most *MLO* genes in the R material showed significantly downregulated expression compared to the S material, including *Moc10g30350.1*, *Moc02g03650.1*, *Moc04g35620.1*, *Moc01g29730.1*, *Moc03g20150.1*, *Moc01g32020.1*, and *Moc09g35890.1* (Figure 9C). Furthermore, it was found that during the PM outbreak period (third and fourth days) in the S material, *Moc10g30350.1* exhibited significantly lower expression in R material (Figure 9C,D), which might be involved in regulating PM resistance in the R material.

### 3.7. qRT-PCR Validation

**Figure 10:**
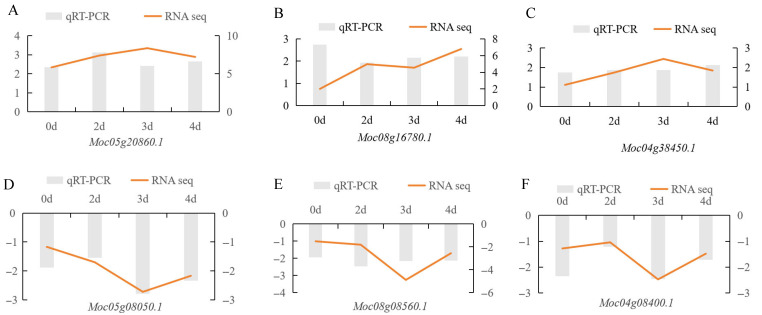
Validation of RNA-seq by qRT-PCR, using 6 DEGs (*Moc05g20860.1*, *Moc08g16780.1*, *Moc04g38450.1*, *Moc05g08050.1*, *Moc08g08560.1*, and *Moc04g08400.1*).

To validate the accuracy of RNA-seq data, six DEGs (*Moc05g20860.1*, *Moc08g16780.1*, *Moc04g38450.1*, *Moc05g08050.1*, *Moc08g08560.1*, and *Moc04g08400.1*) were selected for qRT-PCR analysis (Figure 10). Results showed that three genes (*Moc05g20860.1*, *Moc08g16780.1*, and *Moc04g38450.1*) that were significantly upregulated in the R material by RNA-seq were also significantly upregulated in qRT-PCR (Figure 10A–C). Similarly, three genes (*Moc05g08050.1*, *Moc08g08560.1*, and *Moc04g08400.1*) that were significantly downregulated in the R material by RNA-seq were significantly downregulated in qRT-PCR (Figure 10D–F). The expression trends of these DEGs in qRT-PCR were consistent with RNA-seq results, which fully demonstrated the repeatability of the RNA-seq data.

## 4. Discussion

PM is a devastating fungal disease, which is a major factor affecting yields in cucurbit crops such as bitter gourd [27], cucumber [40], watermelon [41], and pumpkin [42]. PM infection could also reduce photosynthetic efficiency, induce leaf chlorosis and necrosis, disrupt metabolite synthesis, and, finally, affect fruit’s appearance and nutritional content [43]. As a member of the cucurbit crops, bitter gourd is mainly cultivated in tropical and subtropical regions, where it is frequently affected by PM. It is important to investigate the resistance mechanisms against PM in bitter gourd for disease-resistance breeding. In this study, physiological races of PM pathogen affecting bitter gourd were identified through morphological and molecular characterization, and phenotypic changes and RNA-seq analysis were conducted in two bitter gourd cultivars, which could provide a foundation for resistance breeding in bitter gourd.

From a taxonomic perspective, PM pathogen covered 18 genera and 873 species, including *Podosphaera*, *Erysiphe*, and *Golovinomyces* [8]. Due to evolutionary adaptation, pathogenic gene mutations, and horizontal gene transfer, unique physiological races could emerge in different geographical regions [12]. As a result of differences in PM pathogen physiological races, hosts exhibit distinct resistance levels to different PM physiological races [44]. The accurate identification of PM pathogens and their physiological strains would be critical for the effective management of PM. In this study, a comprehensive approach combining microscopic observation and molecular identification was employed to characterize the PM pathogen infecting bitter gourd. Morphological analysis under light microscopy revealed typical PM characteristics: transparent, oval conidia with septate, and branched hyphae. Molecular identification results showed that the PM pathogen in bitter gourd shared 100% homology with reference sequences (MT250855.1 and MH084745.1), which was classified into the *P. xanthii* clade. These findings confirmed that *P. xanthii* was the pathogen responsible for PM disease in bitter gourd.

The response process of plants to *P. xanthii* infection is highly complex and regulated by multiple factors [45]. In this study, two bitter gourd materials (susceptible material S and resistant material R) were used. Phenotypic analysis at different periods after *P. xanthii* infection revealed no obvious disease symptoms in either material during 0–2 d. Therefore, RNA-seq was performed at four periods (0 d, 2 d, 3 d, and 4 d) to evaluate their responses to PM. Transcriptome results revealed that the number of DEGs significantly increased on the third day compared to the second day with the *P. xanthii* infection, which might be attributed to the emergence of obvious PM symptoms in the susceptible material S on the third day. GO enrichment analysis revealed that DEGs on the second, third, and fourth days after *P. xanthii* infection were significantly enriched in pathways including response to stimulus, response to stress, response to biotic stimulus, defense response, and cell wall biogenesis. These GO pathways were consistent with the previous findings in melon [27] and cucumber [46] after PM inoculation. KEGG enrichment analysis revealed that plant–pathogen interaction pathways were significantly enriched after *P. xanthii* infection. As powdery mildew spots increased in susceptible material S, photosynthesis-related pathways were significantly inhibited, and photosynthesis-related gene expression was significantly downregulated. As key components of plant–pathogen interaction pathways, *CaM* and *CML* could participate in plant growth and development as well as various stress responses, including the regulation of plant disease resistance [17]. In this study, the DEGs of *CaM* and *CML* were found to be numerous during the PM onset period in susceptible material S. Notably, both the *CaM* and *CML* genes were significantly upregulated in resistant material, which indicated that the calcium signaling pathway could act as a positive regulator of PM disease resistance. The SEM analysis of the R at 3–4 days revealed inhibited fungal hyphal elongation. During this period, transcriptomic enrichment in cell wall biogenesis, response to fungus, the MAPK signaling pathway, and plant–pathogen interactions directly suppressed fungal growth by strengthening the cell wall barrier, synthesizing antimicrobial substances, and regulating defense gene expression.

A number of studies have demonstrated that TFs could participate in the regulation of plant responses to various pathogenic microorganisms by activating different signaling pathways [47]. Among them, TFs such as *WRKY* [48], *NAC* [49], *ERF* [50], and *bHLH* [18] have been reported in multiple crop disease resistance studies. In this study, expression analysis revealed significant expression differences in *MYB*, *bHLH*, and *ERF* TFs between the R and S materials after *P. xanthii* infection. Among them, one *bHLH* transcription factor (*Moc06g04560.1*) showed significant upregulation in the R resistant material, which might be involved in regulating PM resistance in plants. For example, overexpression of the *CmbHLH87* gene in pumpkin significantly enhanced PM resistance [18]. Concurrently, *ERF* could participate in regulating plant disease responses through the MAPK signaling pathway plants, and overexpression of *ERF* gene could enhance plants’ disease resistance. In this study, two ERF transcription factors (*Moc04g32470.1* and *Moc01g34750.1*) were significantly upregulated in R materials after *P. xanthii* infection, which might be related to the regulation of PM resistance in plants.

As one of the conserved gene families in plants, *MLO* plays a crucial role in plant development and disease resistance [23]. Silencing or knocking out specific *MLO* genes could confer PM resistance in various crops, including barley [51], wheat [22], and grapes [52]. For example, knocking out the *CsMLO1*/*8*/*11* genes from the *CsMLO* family in cucumber could result in 100% resistance to PM [53]. In this study, a total of 15 *MLO* genes were identified, which was consistent with the previous results [54]. RNA-seq analysis revealed that *MLO* genes were predominantly downregulated in the resistant material R. On the third day, multiple *MLO* genes were significantly downregulated in the resistant material R, including *Moc10g30350.1*, *Moc02g03650.1*, *Moc04g35620.1*, *Moc01g29730.1*, *Moc03g20150.1*, *Moc01g32020.1*, and *Moc09g35890.1*, which was similar to the melon research [55]. Furthermore, it was found that *Moc10g30350.1* was significantly downregulated in the R material on both the third and fourth days, which might be related to regulating bitter gourd PM resistance. Homology analysis revealed that *Moc10g30350.1* had a high sequence similarity to *Csa6G509690.1* and *MELO3C007979.1* in cucurbit *MLO* genes (Figure S3). Previous research showed that downregulation of the homologous gene *Csa6G509690.1* could enhance PM resistance in plants [56]. This result suggested that the *Moc10g30350.1* gene could potentially regulate PM resistance in plants. Therefore, further investigation of *Moc10g30350.1* in bitter gourd is necessary to clarify its functional role in regulating the PM resistance response.

## 5. Conclusions

In this study, morphological and molecular analyses identified the PM pathogen in bitter gourd as the physiological race *Podosphaera xanthii*. Scanning electron microscopy of leaves at different periods after inoculation revealed growth differences in the *P. xanthii* among various disease-resistant materials. RNA sequencing technology revealed multiple regulatory pathways governing plant resistance to PM disease. Analysis of transcription factor expression at different periods suggested that a *bHLH* transcription factor (*Moc06g04560.1*) might participate in PM defense. Additionally, analysis of *MLO* gene expression at different periods indicated that an *MLO* gene (*Moc10g30350.1*) may also be involved in PM defense. These findings not only provide a foundation for future bitter melon disease resistance research but also offer new insights for breeding PM-resistant bitter gourd varieties.

## Figures and Tables

**Table 1:** Statistics of transcriptome data in resistant cultivar, R, and the susceptible cultivar, S, leaves after *P. xanthii* infection.

Sample	Raw Bases	Clean Bases	Q20 Rate	Q30 Rate	Total Mapped (%)	Unique Mapped (%)	Multiple Mapped (%)	Note
S1-1	6,422,622,599	6,422,621,067	99.59%	98.14%	95.29	93.32	1.97	S leaves from 0 d after *P. xanthii* infection, replicate 1
S1-2	6,406,034,319	6,406,032,606	99.57%	98.09%	95.45	92.94	2.51	S leaves from 0 d after *P. xanthii* infection, replicate 2
S1-3	6,423,245,294	6,423,244,200	99.55%	98.00%	95.05	91.9	3.15	S leaves from 0 d after *P. xanthii* infection, replicate 3
S2-1	5,949,178,756	5,949,178,030	99.52%	98.20%	97.08	91.08	6	S leaves from 2 d after *P. xanthii* infection, replicate 1
S2-2	5,660,981,999	5,660,981,348	99.53%	98.22%	94.83	89.39	5.44	S leaves from 2 d after *P. xanthii* infection, replicate 2
S2-3	6,046,311,957	6,046,311,577	99.51%	98.14%	97.43	91.38	6.05	S leaves from 2 d after *P. xanthii* infection, replicate 3
S3-1	6,379,142,585	6,379,142,095	99.57%	98.12%	94.72	88.63	6.09	S leaves from 3 d after *P. xanthii* infection, replicate 1
S3-2	6,398,157,856	6,398,157,391	99.56%	98.09%	93.32	88.19	5.13	S leaves from 3 d after *P. xanthii* infection, replicate 2
S3-3	6,424,807,168	6,424,806,843	99.58%	98.14%	95.52	89.4	6.12	S leaves from 3 d after *P. xanthii* infection, replicate 3
S4-1	6,387,274,092	6,387,273,654	99.59%	98.22%	93.46	87.91	5.55	S leaves from 4 d after *P. xanthii* infection, replicate 1
S4-2	6,415,222,101	6,415,221,598	99.57%	98.12%	93.19	87.91	5.28	S leaves from 4 d after *P. xanthii* infection, replicate 2
S4-3	6,428,912,939	6,428,912,577	99.55%	98.04%	93.13	86.26	6.87	S leaves from 4 d after *P. xanthii* infection, replicate 3
R1-1	5,844,186,424	5,844,186,118	99.41%	97.80%	91.97	84.42	7.55	R leaves from 0 d after *P. xanthii* infection, replicate 1
R1-2	6,079,347,055	6,079,346,832	99.48%	98.04%	92.82	82.24	10.58	R leaves from 0 d after *P. xanthii* infection, replicate 2
R1-3	6,393,222,007	6,393,221,515	99.68%	98.65%	89.43	81.07	8.36	R leaves from 0 d after *P. xanthii* infection, replicate 3
R2-1	5,929,993,595	5,929,993,216	99.60%	98.35%	91.98	83.91	8.07	R leaves from 2 d after *P. xanthii* infection, replicate 1
R2-2	6,444,837,279	6,444,837,035	99.69%	98.68%	91.53	82.26	9.27	R leaves from 2 d after *P. xanthii* infection, replicate 2
R2-3	6,423,554,130	6,423,553,643	99.61%	98.24%	92.91	82.94	9.97	R leaves from 2 d after *P. xanthii* infection, replicate 3
R3-1	6,427,115,185	6,427,114,836	99.67%	98.61%	91.31	80.98	10.33	R leaves from 3 d after *P. xanthii* infection, replicate 1
R3-2	6,205,242,855	6,205,242,397	99.61%	98.48%	91.96	82.98	8.98	R leaves from 3 d after *P. xanthii* infection, replicate 2
R3-3	6,444,806,901	6,444,806,713	99.59%	98.18%	95.96	85.96	10	R leaves from 3 d after *P. xanthii* infection, replicate 3
R4-1	6,412,320,897	6,412,320,493	99.63%	98.33%	94.09	85.48	8.61	R leaves from 4 d after *P. xanthii* infection, replicate 1
R4-2	6,441,096,616	6,441,096,338	99.56%	98.08%	91.8	86.13	5.67	R leaves from 4 d after *P. xanthii* infection, replicate 2
R4-3	6,443,186,760	6,443,186,330	99.59%	98.23%	91.37	87.15	4.22	R leaves from 4 d after *P. xanthii* infection, replicate 3

## Data Availability

The RNA-seq data has been submitted to NCBI SRA: PRJNA1393243. The relevant data are available in the Supplementary Materials.

## Supplementary Materials

The following supporting information can be downloaded at: https://www.mdpi.com/article/10.3390/jof12010080/s1, Figure S1. Heat map and qRT- PCR of DEGs in plant hormone signaling transduction pathways between R and S at 2d, 3d and 4d responding to *P*. *xanthii* infection. Figure S2. Heat map of DEGs in plant-pathogen interaction pathway between R and S at 0d responding to *P*. *xanthii* infection. Figure S3. Homology alignment analysis of *Moc10g30350.1*, *Csa6G509690.1* and *MELO3C007979.1* in cucurbit *MLO* genes. Table S1. The primers used in this study. Table S2. Differential expression analysis and functional annotation of genes between S and R on 0 d after *P. xanthii* infection. Table S3. Differential expression analysis and functional annotation of DEGs between S and R on 2 d after *P. xanthii* infection. Table S4. Differential expression analysis and functional annotation of DEGs between S and R on 3 d after *P. xanthii* infection. Table S5. Differential expression analysis and functional annotation of DEGs between S and R on 4 d after *P. xanthii* infection.

## Abbreviations

The following abbreviations are used in this manuscript:

PM	Powdery mildew
DEGs	Differentially expressed genes
TF	Transcription factor
PTI	Pathogen-associated molecular pattern-induced immunity
ETI	Effector-induced immunity
SEM	Scanning electron microscopy
CML	Calcium-binding proteins
CaM	Calmodulin
FLS2	Serine/threonine protein kinase
*RPM1*	Plant immune receptor protein
*CNGC**s*	Cyclic nucleotide-gated channels
