# Supplementary material for: Morphology and Comparative Transcriptome Analysis of Resistant and Susceptible Bitter Gourd (Momordica charantia L.) Reveals the Molecular Response Related to Powdery Mildew Resistance
Source: J Fungi (Basel). 2026 Jan 22;12(1):80. doi: 10.3390/jof12010080 (PMC12843124; doi:10.3390/jof12010080)
Supplement: Supplementary file 1 [file jof-12-00080-s001.zip › Supplementary figures.pdf]

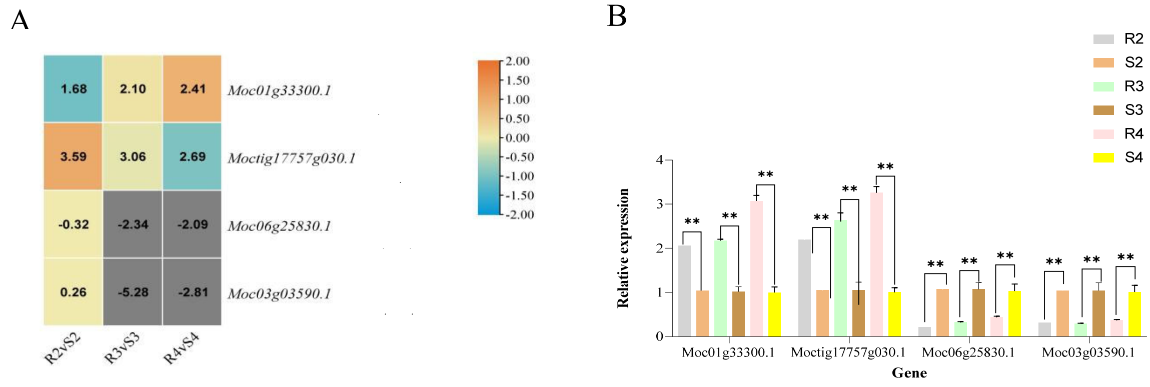

Figure S1. Heat map and qRT- PCR of DEGs in plant hormone signaling transduction pathways between R and S at 2d, 3d and 4d responding to *P. xanthii* infection.(A) Heat map of DEGs in plant hormone signaling transduction pathways between R and S at 2d, 3d and 4d responding to *P. xanthii* infection, the values in the heatmap represented the log_2FoldChange values of genes in R compared to S; (B) qRT- PCR of DEGs in plant hormone signaling transduction pathways between R and S at 2d, 3d and 4d responding to *P. xanthii* infection. Two-tailed Student' s t-test was used to analyze the significance of differences. *P value* < 0.05 (*) and *P value* < 0.01 (**) are regarded as significant.

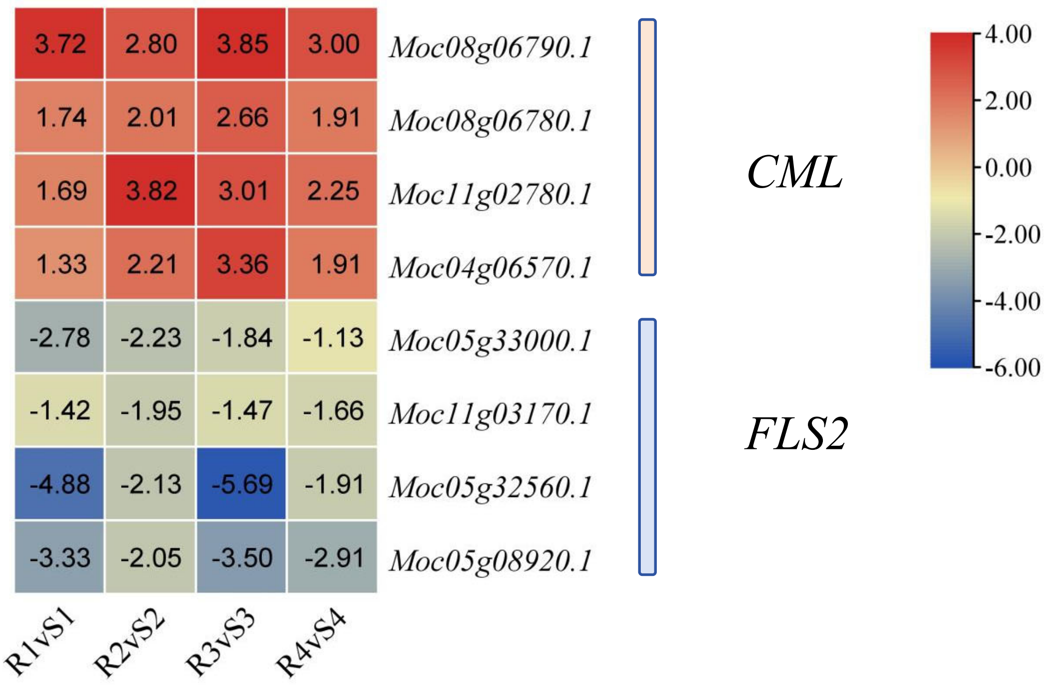

Figure S2. Heat map of DEGs in plant-pathogen interaction pathway between R and S at 0d responding to *P. xanthii* infection; The values in the heatmap represented the $\log_2$FoldChange values of genes in R compared to S.

```
Moc10g30350.1     ..MSVFCLFLCLLFGGAALVAASGVGDSRELDSTPTWAVAGVSFFFIVISIVLEKVLHKLGTWLADKHKSALYEALEKVKAELMILGFISLLLTFAQKYIVQICIPPAVANTMLPCPLEE   118
MELO3C007979.1    ..MSVFCLFLCLLFGGAALVAASGVGDSRELDSTPTWAVAGVSFFFIVISIVLEKVLHKLGTWLADKHKSALYEALEKVKAELMILGFISLLLTFAQKYIVQICIPPAVANTMLPCPLEE   118
Csa6G509690.1     MLLVVYYLCLSLLWG.KSWGAPASDGTTRELDQTPTWAVAGVCAIIILISIALEKLLHKAGTWLTEKHKRALFEALEKVKAELMILGFISLLLTFGQNYIIKICIPTKVANTMLPCAAKE   119
Consensus           msvfclflcllfggaalvaasgvgdsreldstptwavagvsfffivisivlekvlhklgtwladkhksalyealekvkaelmilgfislllltfaqkyivqicippavantmlpcplee

Moc10g30350.1     KDAPSSLAGEEEHHRRLQRLNRRSLAGGHDVVSCKDGKVSLISVDGLHQLHILIFFLAVFHVLFCVTTMTLGRIKARGWKEWEQETSTHNYEFFNDPARFRLTHETSFVKAHTSFWTRLP   238
MELO3C007979.1    KDAPSSLAGEEEHHRRLQRLNRRSLAGGHDVVSCKDGKVSLISVDGLHQLHILIFFLAVFHVLFCVTTMTLGRIKARGWKEWEQETSTHNYEFFNDPARFRLTHETSFVKAHTSFWTRLP   238
Csa6G509690.1     DKLEK..GDEGEHHRRLLMYERRFLAAGGAVSCKEGHVPLISISGLHQLHLFIFFLAVFHVVYSAITMMLGRLKIRGWKAWEEETSTHNYEFSN...............AHTSFWTKLP   222
Consensus         kdapsslageeehhrrlqrlnrrslagghdvvsckdgkvslisvdglhqlhilifflavfhvlfcvttmtlgrikargwkeweqetsthnyeffndparfrlthetsfvkahtsfwtrlp

Moc10g30350.1     FFFYIACFFRQFYGSVSKADYLTLRNGFITVHLGPGSKFNFRRYIKRSLEDDFKVVVGVSPVLWLSFVIFLLLNVAGWRTLFWASFTPLIIIILAVGSKLQAILTRMALEISERHAVVQGI   358
MELO3C007979.1    FFFYIACFFRQFYGSVSKADYLTLRNGFITVHLGPGSKFNFRRYIKRSLEDDFKVVVGVSPVLWLSFVIFLLLNVAGWRTLFWASFTPLIIIILAVGSKLQAILTRMALEISERHAVVQGI   358
Csa6G509690.1     VFFYIGCFFRQFFKSVGKADYLALRNGFIAVHLAPGSKFDFQKYIKRSLEDDFKIIVGVSPVLWTSFVVFLLINVYGWQALFWSSLVPVIIILAVGTKLQGVMTKMALEITERHAVVQGI   342
Consensus         fffyiacffrqfygsvskadyltlrngfitvhlgpgskfnfrryikrsleddfkvvvgvspvlwlsfvifllllnvagwrtlfwasftpliiiilavgsklqailtrmaleiserhavvqgi

Moc10g30350.1     PLVQGSDKYFWFGRPQLILHLMHFALFQSAFQTTFILSTMHSFGPKSCFSDGVFLTSLKVVLGFAALFLCSYVTLPIYALVTQMGSGMKKSVFDEQTSKALKRWRDTVKKKRVQRASATQ   478
MELO3C007979.1    PLVQGSDKYFWFGRPQLILHLMHFALFQSAFQTTFILSTMHSFGPKSCFSDGVFLTSLKVVLGFAALFLCSYVTLPIYALVTQMGSGMKKSVFDEQTSKALKRWRDTVKKKRVQRASATQ   478
Csa6G509690.1     PLVQASDKYFWFGKPQLVLYLIHFALFSNAFQITYFFWIWYSFGLKSCFHTDFKLAIIKVGLGVGVLCLCSYITLPLYALVTQMGTRMKKSIFDEQTSKALKKWHMAVKKR..HGKSPTR   460
Consensus         plvqgsdkyfwfgrpqlilhlmhfalfqsafqttfilstmhsfgpkscfsdgvfltslkvvlgfaalflcsyvtlpiyalvtqmgsgmkksvfdeqtskalkrwrdtvkkkrvqrasatq

Moc10g30350.1     TLGGGSNASPVRSLGRSLRRFKTTGHSIRVDAYEDLESSDYEGDALATPAPESINVDVRGEEIQQVVETNQAHLVEQTEEEGDESSFIKAAATAAIATP   577
MELO3C007979.1    TLGGGSNASPVRSLGRSLRRFKTTGHSIRVDAYEDLESSDYEGDALATPAPESINVDVRGEEIQQVVETNQAHLVEQTEEEGDESSFIKAAATAAIATP   577
Csa6G509690.1     KLGSPSSSPIHPSSGYALHRFKTTGHSNRSSMYDENDASDYEVDTPN....FTVRIDHGDEHQAEIIEP..QHTEKRNE...DDFSFVKPGPTK.....   545
Consensus         tlgggsnaspvrslgrslrrfkttghsirvdayedlessdyegdalatpapesinvdvrgeeiqqvvetnqahlveqteeegdessfikaaataaiatp
```

Figure S3. Homology alignment analysis of *Moc10g30350.1*, *Csa6G509690.1* and *MELO3C007979.1* in cucurbit MLO genes
